# LPS-Induced TNF-α and IL-1 Signaling in Periodontitis: Molecular Mechanisms and Therapeutic Opportunities

**DOI:** 10.3390/cimb48090889

**Published:** 2026-08-31

**Authors:** Maedeh Vakili Saatloo, Serge Dibart, Yun Ma

**Affiliations:** Department of Periodontology, Boston University Henry M. Goldman School of Dental Medicine, 635 Albany Street, Boston, MA 02118, USA; maedehvs@bu.edu (M.V.S.); sdibart@bu.edu (S.D.)

**Keywords:** periodontitis, inflammation, TNF-α, IL-1β, lipopolysaccharide, LPS, molecular mechanisms, therapeutics

## Abstract

Periodontitis is a chronic, dysbiosis-driven inflammatory disease, that, unlike gingivitis, results in irreversible, tooth-supporting soft tissue and bone loss. Clarifying the molecular pathways that sustain the host inflammatory response is therefore central to improving disease management. Lipopolysaccharide (LPS), a major pathogen-associated molecular pattern of Gram-negative bacteria, is one important—though not the sole—microbial stimulus that drives inflammation in the polymicrobial, dysbiotic periodontal environment. This narrative review summarizes how LPS-initiated signaling promotes production of the pro-inflammatory cytokines TNF-α and IL-1, how these cytokines couple inflammation to osteoclast-mediated bone loss, and whether their pharmacological blockade holds therapeutic promise. A literature search combining the terms “LPS,” “periodontitis,” “TNF-α,” and “IL-1” was conducted in PubMed/MEDLINE, Scopus, and Google Scholar for English-language articles published up to 2026, with priority given to primary studies over secondary citations. Preclinical models consistently show that TNF-α and IL-1 blockade reduces periodontal inflammation and bone loss, but the effect is time-dependent: short-term blockade can aid healing whereas prolonged blockade may impair it, and robust periodontitis-specific clinical trials are lacking. We conclude that targeted modulation of TNF-α and IL-1—particularly through locally delivered, appropriately timed agents used as an adjunct to conventional mechanical therapy—is a biologically rational strategy.

## 1. Introduction

Periodontitis is a chronic inflammatory disease and develops in response to a dysbiotic subgingival biofilm, leading to progressive destruction of the periodontal soft tissue and alveolar bone and, if untreated, to tooth loss. It is highly prevalent, affecting nearly half of adults worldwide, with roughly 10% experiencing severe forms [1,2]. Beyond the oral cavity, periodontitis is increasingly recognized as a contributor to systemic inflammatory burden, and epidemiological and mechanistic studies have linked it to cardiovascular disease [3,4,5,6], Alzheimer’s disease (AD) [7], Parkinson’s disease [8,9], type 2 diabetes mellitus (in a bidirectional relationship) [10], and rheumatoid arthritis (RA) [11]. TNF-α and IL-1 are implicated both locally and as mediators of this systemic crosstalk, which is one reason that dampening their activity could produce benefits extending beyond the periodontium [12,13,14,15].

The etiology of periodontitis is microbial, but the tissue destruction is driven by the host inflammatory response to microbial challenge [16]. The dysbiotic subgingival biofilm is characterized by a high abundance of Gram-negative bacteria. Among them, *Porphyromonas gingivalis* (*P. gingivalis*) acts as a keystone pathogen [17,18], orchestrating dysbiosis and altering host-microbial homeostasis to promote disease progression [19]. *P. gingivalis* has been detected in approximately 85.75% of subgingival plaque samples from periodontitis patients, and its abundance correlates with disease severity [20]. *P. gingivalis* and other Gram-negative bacteria possess LPS and various virulence factors, including gingipains, fimbriae, nucleic acids, and lipoproteins, which function as pathogen-associated molecular patterns (PAMPs). These PAMPs modulate host immune responses and thereby contribute to the pathogenesis of periodontitis [21]. LPS acts as a prominent inflammatory stimulus within this complex microbial community. This review focuses on LPS-initiated signaling because it is among the best-characterized routes to TNF-α and IL-1 induction, while keeping this broader context in view.

Conventional management of periodontitis rests on mechanical disruption of the biofilm (scaling and root planning, surgical access where needed) and, in selected cases, adjunctive antimicrobials. These approaches are effective for many patients but have well-recognized limitations: residual deep pockets, disease recurrence, a subset of “non-responding” patients, and—critically—an incomplete effect on the host inflammatory response that drives tissue breakdown. Persistent inflammation, ongoing bone destruction, and heterogeneous responses to standard therapy together motivate interest in host-modulation strategies that target the inflammatory mediators themselves [22,23,24,25,26,27].

Building on the clinical success of cytokine blockade in RA, psoriasis, and inflammatory bowel disease, we examine whether neutralizing TNF-α or IL-1 could similarly benefit the periodontium. The central hypothesis motivating this review is that targeted modulation of TNF-α and IL-1 signaling may serve as an adjunctive strategy—complementing, not replacing, mechanical biofilm control—to reduce periodontal inflammation and preserve the tooth-supporting tissues. We first summarize LPS structure and its receptor-level recognition; then trace the downstream signaling that generates TNF-α and IL-1 and couples them to bone loss; and finally appraise the preclinical and (limited) clinical evidence for anti-TNF-α and anti-IL-1 therapy, giving particular weight to the questions of delivery route, treatment timing, safety, and evidence quality that will determine any future translational path.

## 2. Materials and Methods

A literature search was conducted in PubMed/MEDLINE, Scopus, and Google Scholar for English-language articles published from 1980s up to July 2026. using combinations of keywords: “periodontitis”, “inflammation”, “lipopolysaccharide”, “LPS”, “TNF-α”, “IL-1β”, “IL-1”, “TNF/IL-1 inhibitor/biologic therapy”. Reference lists of relevant articles were hand-searched for additional primary sources. Records were screened manually in three sequential steps: (1) studies had to be relevant to LPS, TNF-α, or IL-1 in the context of periodontitis; (2) priority was given to original research over review articles that merely cited primary results; and (3) referenced findings were checked against the primary source. Because a narrative review does not apply a formal risk-of-bias assessment, we instead sought to signpost the level of evidence throughout the text—distinguishing in vitro studies, animal models, human observational studies, and controlled clinical trials—so that preclinical findings are not given the same interpretive weight as clinical evidence. The figures included in this article were created by the authors using “PowerPoint” with some basic shapes derived from “Servier Medical Art, licensed under Creative Commons Attribution 4.0 International (CC BY 4.0)”.

## 3. LPS—TNF-α/IL-1 Signaling in Periodontitis

### 3.1. LPS Structure and Its Immune-Stimulatory Function

#### 3.1.1. LPS Structure

**Figure 1:**
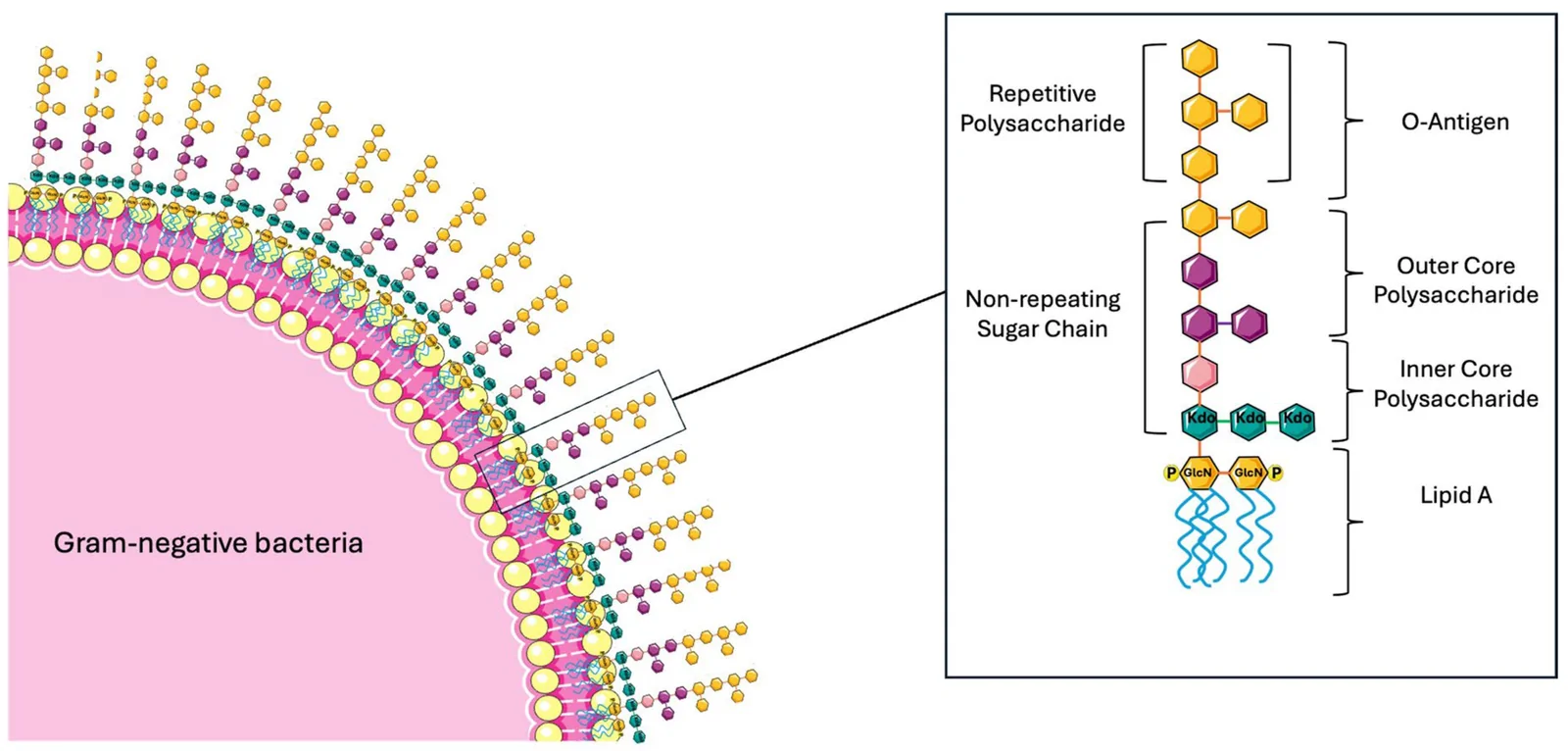
Schematic of the Gram-negative outer membrane and LPS structure.

LPS is an integral component of Gram-negative bacteria outer membrane [28] and is composed of three main elements: (1) lipid A; (2) core oligosaccharide; and (3) the O antigen (Figure 1). The structure and composition of these elements vary across and within bacterial species [29], and this variation produces diverse host immune responses. For instance, the lipid A of *Escherichia coli* (*E. coli*) is highly immunogenic, whereas other lipid A structures are far less so; producing weakly immunogenic lipid A is one strategy used by certain bacteria to evade immune detection and persist without triggering strong activation [30].

#### 3.1.2. Recognition of LPS by Toll-like Receptors

LPS triggers immune responses chiefly through its lipid A moiety, which is recognized by the host’s Toll-like receptors (TLRs) [31]. TLRs are pattern-recognition-receptors (PRRs) that detect conserved microbial structures; human express ten TLRs, whereas mice express thirteen. TLR activation has been implicated in inflammatory disease across many organs, including the oral cavity, where it contributes to periodontal inflammation and alveolar bone loss [32]. TLR4 primarily detects LPS from Gram-negative bacteria whereas TLR2 recognizes lipoteichoic acid (LTA) from Gram-positive bacteria as well as certain bacterial lipoproteins [33]; both TLR2 and TLR4 predominate in periodontal tissues [34,35,36,37].

A crucial and disease-relevant point is that the LPS of *P. gingivalis* is atypical. Unlike the relatively uniform LPS of enteric bacteria, *P. gingivalis* produces a heterogeneous mixture of lipid A species: tetra-acylated forms act as weak TLR4 agonists or even TLR4 antagonists, whereas penta-acylated forms activate TLR4-dependent signaling [38]. This structural flexibility is a core strategy by which *P. gingivalis* manipulates host immunity, and it underscores why LPS-driven signaling in periodontitis cannot be treated as a single, uniform input. In human gingival fibroblasts and macrophages, different *P. gingivalis* lipid A structures differentially alter NF-κB activation and the production of TNF-α, IL-1β, IL-6, and IL-8 [38].

#### 3.1.3. Downstream Signaling from LPS-TLR Engagement

**Figure 2:**
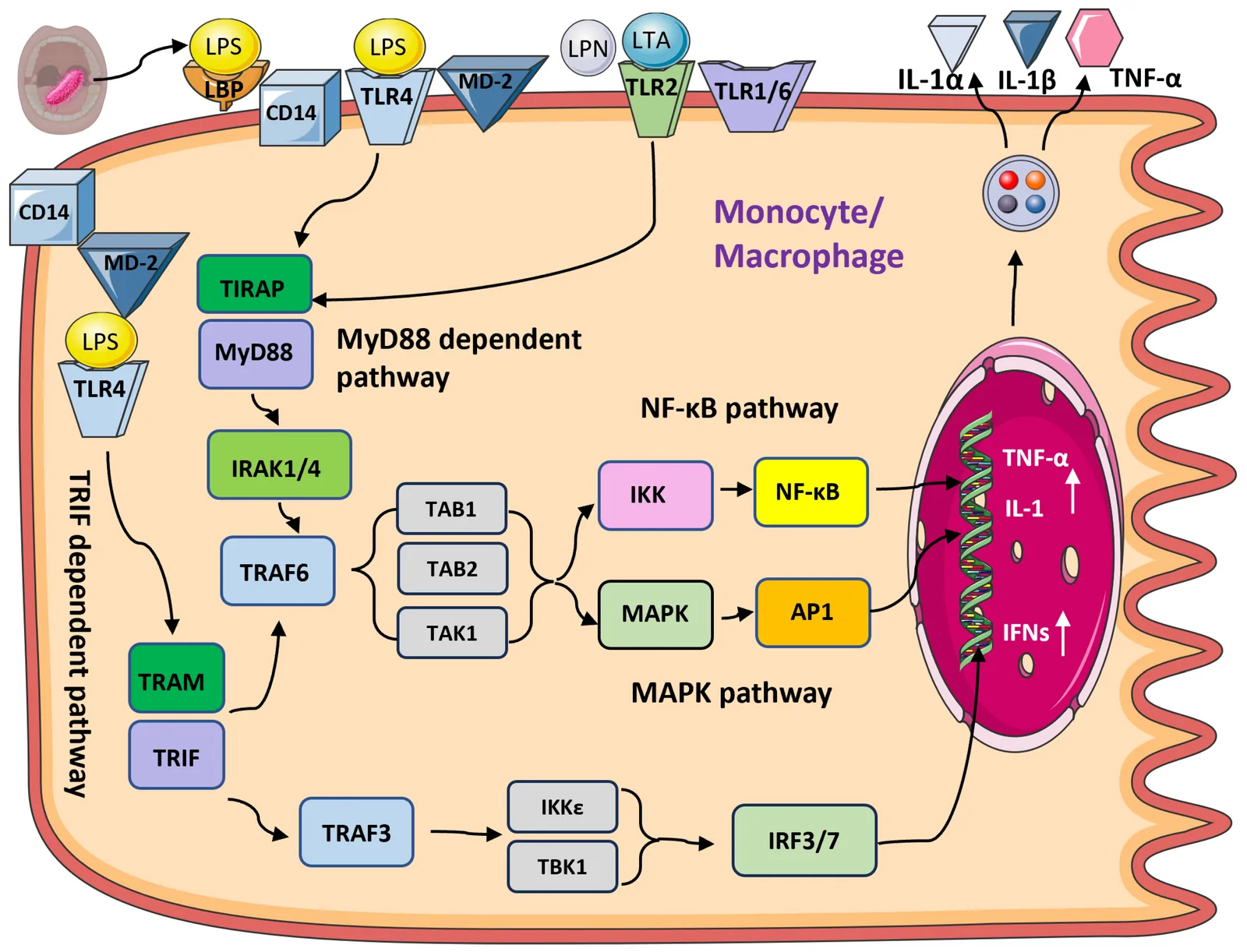
Binding of LPS to TLR4 (with TLR2) activates the MyD88-dependent pathway, and, after receptor internalization, the TRIF-dependent (MyD88-independent) pathway; MyD88-dependent signaling drives NF-κB/AP-1 and production of IL-1 and TNF-α, whereas the TRIF branch activates IRF3/7 and type I IFNs. LPS: lipopolysaccharide; LBP: LPS binding protein; TLR: toll-like receptor; CD14: cluster of differentiation 14; MD-2: myeloid differentiation factor 2; LTA: lipoteichoic acid; LPN: lipoprotein; TIRAP: TIR domain-containing adaptor protein; MyD88: myeloid differentiation primary response 88; IRAK1/4: interleukin-1 receptor-associated kinase 1/4; TRAF: TNF receptor-associated factor; TAB: TAK-binding protein; TAK1: TGF-β-activated kinase 1; IKK: inhibitor of κB kinase; NF-κB: nuclear factor kappa-light-chain-enhancer of activated B cells; MAPK: mitogen-activated protein kinase; AP1: activator protein-1; TNF-α: tumor necrosis factor-alpha; IL-1: interleukin-1; TRAM: TRIF-related adaptor molecule; TRIF: TIR-domain-containing adaptor-inducing beta interferon; TBK1: TANK-binding kinase 1; IRF: interferon regulatory factor; IFNs: interferons.

Signals from LPS–TLR engagement are transmitted intracellularly through adaptors and kinases including MyD88, interleukin-1,4 receptor-associated kinases (IRAK1,4), TNF receptor-associated factor 6 (TRAF6), and NF-κβ, leading to production of proinflammatory cytokines [39]. TLR4 signaling is notable in that it bifurcates into two branches (Figure 2). The MyD88-dependent branch, recruited at the plasma membrane via the sorting adaptor TIRAP, activates NF-κB and activator protein-1 (AP-1) to drive early transcription of TNF-α and IL-1 [39]. The TRIF-dependent (MyD88-independent) branch, engaged after TLR4 internalization via the adaptor TRAM, activates interferon regulatory factor 3 (IRF3) to induce type I interferons (IFNs) and also contributes to a later phase of NF-κB activation [40]. Distinguishing these branches matters biologically because IL-1 and TNF-α promote osteoclast differentiation and activity [41], whereas type I IFNs generally inhibit osteoclast formation [42]; the net osteoclastogenic output of TLR4 signaling thus reflects the balance between its MyD88- and TRIF-driven arms. In periodontal tissues, TLR2 signaling (also MyD88-dependent) provides an additional route to NF-κB activation, and *P. gingivalis* can exploit a TLR2–PI3K axis to escape immune clearance while still promoting bone resorption [43].

#### 3.1.4. Post-Transcriptional Regulation of LPS-Induced Cytokine Production

Although LPS-triggered activation of TLRs and transcription factors such as NF-κB is essential for initiating cytokine gene expression, transcription alone does not fully explain the persistently high levels of TNF-α, IL-1β and IL-6 seen in chronic periodontal lesions; post-transcriptional control of mRNA stability also contributes [44]. A well-characterized mechanism is the p38 mitogen-activated protein kinase (MAPK) pathway and its downstream effector MAPK-activated protein kinase 2 (MK2), which stabilizes mRNAs encoding proinflammatory cytokines [44,45]. Recognizing this post-transcriptional layer helps explain both the chronicity of periodontal inflammation and the variable responses observed with cytokine-targeted therapies [44].

### 3.2. LPS and the Host Immune Response

#### 3.2.1. The Periodontal Immune-Cell Landscape

Analyses of gene-expression data (Gene Expression Omnibus) indicate that plasma cells (differentiated B cells), naïve B cells, and neutrophils are enriched in periodontitis lesions, whereas memory B cells, CD4^+^ memory T cells, and resting dendritic cells predominate in healthy periodontal tissue [46]. During inflammation, immune cells release reactive oxygen species (ROS) and proinflammatory cytokines—including TNF-α, IL-1, and prostaglandin E2 (PGE2)—that together promote osteoclast differentiation and bone resorption [47,48]. *P. gingivalis* fragments and virulence factors activate neutrophils and macrophages, driving cytokine release and RANKL-dependent osteoclastogenesis that ultimately causes alveolar bone loss [43].

#### 3.2.2. LPS and Monocytes/Macrophages

Most myeloid-lineage cells express TLR4. LPS binding to TLR4 requires accessory molecules including LPS-binding protein (LBP), cluster of differentiation 14 (CD14), and MD-2, and specifically involves the acyl chains and phosphate groups of lipid A. In organisms such as *E. coli*, hexa-acylated and diphosphorylated lipid A is a potent TLR4 agonist; Conversely, under-acylation or dephosphorylation markedly reduces proinflammatory activity and is a key mechanism for down-regulating inflammation and promoting immune resolution [49]. This structural dependence again illustrates that “LPS” is not a single functional entity.

#### 3.2.3. LPS and Neutrophils

Human neutrophils express TLR1, 2, 4, 5, 6, 7, 8, 9, and 10 but not TLR3 [50]. Unlike monocytes, LPS-activated neutrophils do not express type I IFN or IFN-dependent genes and thus do not mobilize the MyD88-independent/TRIF-dependent pathway [51]. In health, neutrophils continuously migrate through the junctional epithelium into the gingival sulcus to maintain microbial homeostasis. In periodontitis, heightened neutrophil responsiveness and exaggerated neutrophil extracellular trap (NET) formation can damage the gingival barrier, allowing penetration of bacteria and PAMPs and stimulating Th17 cells; resulting in further neutrophil recruitment and tissue damage [52,53,54].

#### 3.2.4. TNF-α and IL-1 Within the Broader Inflammatory Network

Although this review focuses on TNF-α and IL-1, these cytokines act within an integrated inflammatory network rather than in isolation. LPS-initiated signaling and the TNF-α/IL-1 axis intersect with IL-6, the IL-17/IL-23–Th17 pathway, IFN-γ, chemokines, matrix metalloproteinases (MMPs), the RANKL/OPG axis, and the NLRP3/AIM2 inflammasomes to shape the net outcome of connective-tissue degradation and bone loss. TNF-α and IL-1 frequently function as upstream amplifiers within this network, for example by inducing IL-6 and PGE2 and by up-regulating RANKL, which is part of the rationale for targeting them.

### 3.3. TNF-a and IL-1 Mediate LPS-Induced Inflammation and Bone Loss

LPS has been implicated in the pathogenesis of periodontitis through induction of proinflammatory cytokines such as IL-1 and TNF-α [55] and consequent bone resorption [56].

#### 3.3.1. LPS Exposure, Cytokine Production, and Periodontal Destruction

In vivo (animal): Periodontal-pocket injection of LPS caused alveolar bone loss in rats [57]. Wistar rats developed periodontitis and alveolar bone loss accompanied by elevated NF-κB and RANKL after palatal injection of LPS [58]. These two animal studies demonstrated that local LPS exposure induced periodontal inflammation and tissue loss, providing evidence that bacterial LPS contributes to the pathogenesis of periodontitis. Systemic LPS administration combined with ligature placement in Sprague-Dawley rats led to periodontitis and alveolar bone loss associated with increased TNF-α, IL-6, NLRP3, cleaved caspase-1, and RANKL [59].

In vitro: Mouse RAW264.7 macrophages treated with LPS displayed M1 polarization with increased TNF-α, IL-1β, IL-6, NLRP3, and cleaved caspase-1, and ROS [60]. Human THP1 macrophages showed M1 macrophage polarization with increased TNF-α, IL-1β, and IL-6 [61]. Human periodontal ligament stem cells (hPDLSCs) exposed to LPS exhibited increased TNF-α, IL-1β, IL-6, COX2 and iNOS with reduced osteogenesis [62]. Human periodontal ligament cells (hPDLCs) showed increased TNF-α, IL-6, iNOS, COX2 and ROS with decreased alkaline phosphatase (ALP) activity [63]. Collectively, these in vitro studies provided evidence that LPS exposure triggers an inflammatory cascade within various cells of the periodontium.

Altogether, these in vivo and in vitro studies indicate that LPS exposure elicits robust inflammatory responses and contributes to periodontal tissue destruction (Table 1 and Table 2). Because the two tables mix species, LPS sources, and model systems, they are best read as a catalogue of consistent directional effects rather than as directly comparable quantitative results.

#### 3.3.2. Why This Review Focuses on TNF-α and IL-1

We concentrate on TNF-α and IL-1 for several reasons. First, TNF-α and its receptor TNFR1 are expressed in junctional epithelium, and TNF-α induces RANKL expression via TNFR1 and protein kinase A pathway in gingival epithelial cells [71]. TNF-α is elevated in the gingival crevicular fluid (GCF) [72] and serum [73] of patients with chronic periodontitis. Second, IL-1 is produced in the periodontium and elevated at disease sites [74], with increased IL-1β frequently detected in the saliva and GCF of patients [75,76]; applied alone to rat gingiva, IL-1 enhanced inflammation and bone resorption [77]. Third, TNF-α and IL-1 are rapidly induced by LPS, and other mediators such as IL-6 and PGE2 are downstream of them [78,79,80,81,82,83]. Fourth, TNF-α and IL-1 directly or indirectly regulate osteoclastogenesis and bone resorption, linking inflammation to inflammatory bone loss [84]. Fifth, TNF-α and IL-1 blockers are clinically available and effective in other inflammatory diseases such as RA. Sixth, in a *Macaca fascicularis* primate model of periodontitis, IL-1/TNF antagonists reduced osteoclast number and alveolar bone loss, and soluble IL-1/TNF antagonists reduced connective-tissue attachment loss by roughly half [85,86,87]. Other mediators (IL-6, IL-17 and arachidonic acid metabolites) also contribute to periodontal destruction; IL-6, for example, is directly induced by LPS [88,89] and further modulated by feedback within its own family [90,91]. These are acknowledged as part of the network (Section 3.2.4) but are outside this review’s primary focus.

#### 3.3.3. TNF-α Biology and Periodontitis

TNF-α is a 17-kDa cytokine encoded on chromosome 6 near the HLA-B locus of the major histocompatibility complex (MHC), suggesting a link between TNF-α expression and MHC-associated inflammatory or autoimmune disorders [92]. It exists in soluble and membrane-bound forms [93] and signals through two receptors, TNFR1 and TNFR2, which share extracellular homology but differ in their intracellular regions. Monocytes and macrophages are the primary sources. The magnitude of TNF-α production varies among individuals, influenced by polymorphisms within the TNF locus [94], CD14 expression [95], and the presence of other cytokines. Functionally, TNF-α is a pleiotropic mediator, affecting antimicrobial defense, cell growth, differentiation, and immune modulation [96].

Elevated TNF-α has been consistently detected in the serum, gingival tissue, and GCF of patients with periodontitis [72]. Promoter polymorphisms (notably at −308 *G/A* and −863 *C/A*) have been associated with increased susceptibility [97]. Beyond its role as a proinflammatory mediator, TNF-α contributes to bone metabolism by upregulating RANKL in gingival epithelial cells, T cells, and osteoblasts [71,98], and it induces apoptosis in gingival fibroblasts and epithelial cells, aggravating tissue destruction [99]; *P. gingivalis*-induced fibroblast apoptosis was greatly reduced in *TNFR*^−/−^ mice [100].

#### 3.3.4. IL-1 Biology and Periodontitis

The IL-1 family relevant here comprises two agonists, IL-1α and IL-1β, and one natural antagonist, the IL-1 receptor antagonist (IL-1Ra). Both agonists signal through IL-1R1 [101].

IL-1β production is governed by two distinct signals: Signal 1 (priming): LPS–TLR engagement activates NF-κB, inducing transcription of *IL1B* and synthesis of the inactive precursor pro-IL-1β. This step also up-regulates NLRP3. Signal 2 (activation): Conversion of pro-IL-1β to the mature, secreted, biologically active cytokine requires assembly of an inflammasome (e.g., NLRP3 or AIM2) and activation of caspase-1, which cleaves both pro-IL-1β and gasdermin D; the latter forms membrane pores that mediate IL-1β release and pyroptotic cell death. Alternative, caspase-1-independent proteases can also process IL-1β in some contexts [40,102,103,104].

IL-1α is constitutively present in many cell types and released upon cell death and is bioactive in its precursor form without requiring caspase-1 processing [105,106,107].

The human IL1B gene lies on chromosome 2 and contains regulatory polymorphisms; Carriers of the *IL1B* 3953 *T* allele secrete approximately twice as much IL-1β as those with the *CC* genotype [108], and IL-1 gene polymorphisms have been associated with the susceptibility to, and progression of, periodontitis [109,110,111] and peri-implantitis [112].

Functionally, IL-1 shifts bone remodeling toward resorption: IL-1β administration increased osteoclast number and bone resorption while suppressing bone formation in rats [113,114,115,116], inhibited osteoblastic collagen and non-collagenous protein synthesis and ALP expression in vitro [115], and increased matrix metalloproteinase (MMP) expression, contributing to matrix degradation [117,118]. IL-1α exhibits similar effects as IL-1β does in osteoblasts [119]. IL-1β also induces secondary mediators (IL-6, IL-11, M-CSF) that further amplify the pro-inflammatory milieu and potentiate its osteoclastogenic effects [120]. Consistent with a central role, *IL-1*α/β double-knockout mice display greater bone mineral density and fewer osteoclasts than wild-type mice [114], elevated IL-1/IL-1Ra ratio in GCF tracks with disease severity [121]. IL-1Ra deficiency reduces the expression of osteogenic markers and formation of mineralized matrix by osteoblasts. IL-1Ra deficiency worsens infection-driven periodontal bone loss [122].

#### 3.3.5. Osteoclastogenesis: Canonical RANKL-Dependent Versus TNF-/IL-1-Mediated Amplification

**Figure 3:**
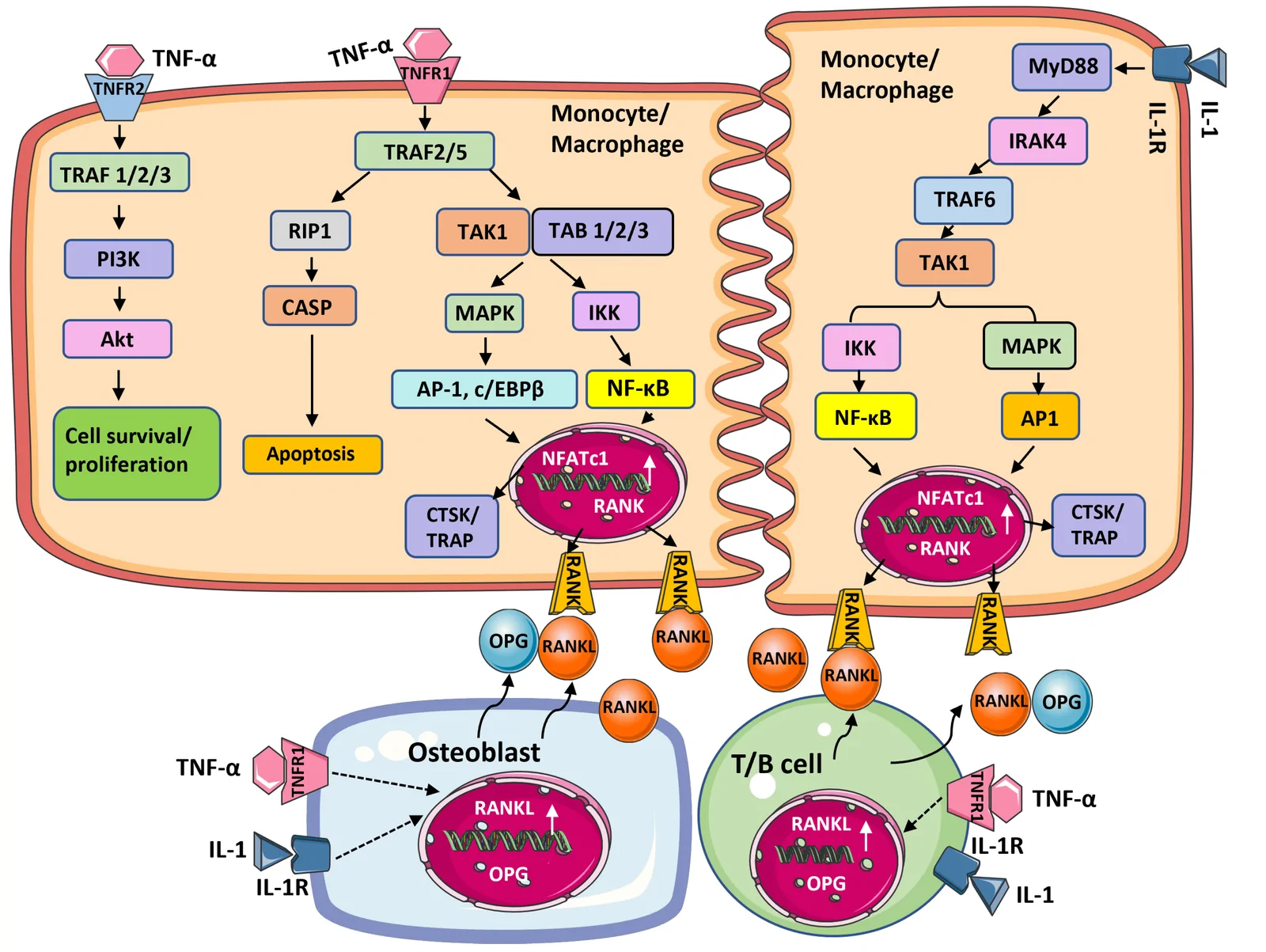
TNF-α/TNFR1 and IL-1/IL-1R signaling amplify canonical RANKL–RANK–NFATc1 osteoclastogenesis—by increasing RANKL and reducing OPG, enhancing precursor survival/fusion, and lowering the RANKL threshold—leading to increased osteoclast formation and activity, whereas the TNF-α and TNFR2 signaling favors cell survival and proliferation. TNF-α: tumor necrosis factor-alpha; TNFR: TNF receptor; IL-1: interleukin-1; IL-1R: IL-1 receptor; MyD88: myeloid differentiation primary response 88; IRAK4: interleukin-1 receptor-associated kinase 4; TRAF: TNF receptor-associated factor; PI3K: phosphoinositide 3-kinase; Akt: protein kinase B; RIP1: receptor-interacting protein 1; CASP: caspase; TAK1: TGF-β-activated kinase 1; TAB: TAK-binding protein; MAPK: mitogen-activated protein kinase; IKK: inhibitor of κB kinase; AP1: activator protein-1; c/EBPβ: CCAAT/enhancer-binding protein beta; NF-κB : nuclear factor kappa-light-chain-enhancer of activated B cells; NFATc1: nuclear factor of activated T cells 1; RANK: receptor activator of nuclear factor kappa-B; RANKL: receptor activator of nuclear factor kappa-B ligand; OPG: osteoprotegerin; CTSK: cathepsin K; TRAP: tartrate-resistant acid phosphatase.

A precise account of how TNF-α and IL-1 drive bone loss requires distinguishing two mechanisms (Figure 3). Canonical osteoclastogenesis is RANKL-dependent: RANKL, presented by osteoblasts, stromal cells, and activated T/B cells, engages RANK on myeloid precursors to induce NFATc1—the master osteoclastogenic transcription factor—and downstream resorptive machinery (cathepsin K, TRAP). TNF-α and IL-1 mainly act to amplify this canonical pathway rather than to substitute for it: they up-regulate RANKL expression by stromal and immune cells, suppress the decoy receptor OPG (shifting the RANKL/OPG balance), enhance the survival and fusion of osteoclast precursors, and lower the RANKL threshold required for differentiation [123,124,125,126]. Under permissive levels of RANKL, TNF-α and IL-1 can further stimulate osteoclast formation and activity [126,127,128], and IL-1 and TNF-α have been reported to support RANKL-independent osteoclast differentiation in specific settings [41]. Framing TNF-α/IL-1 as amplifiers of, and cooperating signals with, RANKL-driven osteoclastogenesis—rather than as direct, standalone inducers of “RANK and NFATc1 production”—is both more accurate and more informative for therapy, because it predicts that cytokine blockade will blunt but not abolish osteoclastogenesis.

### 3.4. TNF-α and IL-1 Antagonism in the Management of Periodontitis

Anti-TNF-α and anti-IL-1 are established treatments for inflammatory and autoimmune diseases such as RA, ankylosing spondylitis, and psoriasis [129,130,131,132]. Because TNF-α and IL-1 contribute substantially to periodontal inflammation and alveolar bone loss, investigators have asked whether blocking TNF-α, IL-1, or both could mitigate periodontal destruction.

#### 3.4.1. Systemic Biologic Therapy—And the Limits of Indirect Evidence

Anti-TNF-α. Infliximab, a TNF-α-neutralizing antibody, reduced gingival myeloperoxidase (MPO), IL-1β, TNF-α, MMP-1/-8, RANK, and RANKL and improved periodontal collagen network in a rat ligature model of periodontitis [133]. Etanercept ameliorated mandibular bone loss in a mouse model of systemic lupus erythematosus (*FcγRIIb*^−/−^) [134]—evidence of anti-resorptive effect, but in an autoimmune model that is not periodontitis, and therefore only indirectly relevant. In RA patients, infliximab treatment reduced periodontal attachment loss but increased gingivitis; the latter was not associated with more periodontal disease [135]. Anti-TNF-α therapy increased the effectiveness of non-surgical periodontal therapy in patients with RA and periodontitis [136]. A separate report cautioned that anti-TNF-α therapy might increase osteonecrosis risk in patients with RA and periodontitis [137].

Anti-IL-1. IL-1Ra (Anakinra) significantly reduced IL-1β-mediated leukocyte clustering and osteoclastogenesis in co-cultures of human periodontal ligament fibroblasts and peripheral blood mononuclear cells [138]; and in IL-1Ra-knockout macrophages, *Aggregatibacter actinomycetemcomitans*-LPS markedly increased IL-1α, IL-1β, TNF-α, and IL-6 with enhanced osteoclast formation [139]. Despite clear anti-inflammatory effects, Anakinra’s rapid renal clearance and short half-life limit its utility, motivating delivery strategies to extend its action [140,141,142,143].

Interpretive caution (Table 3). Several entries in Table 3 are not direct periodontitis- therapy models—for example, the *FcγRIIb*^−/−^ lupus model [134], RA-patient cohorts [135], and psoriasis-comorbidity or diabetic-animal models [144]. These provide supportive, mechanistically consistent, indirect evidence and should not be weighed equally with direct periodontal-intervention studies.

#### 3.4.2. Local and Targeted Delivery as a Distinct Translational Strategy

Systemic biologics carry systemic risk, whereas the periodontal lesion is anatomically accessible and locally confined, which makes local, sustained, site-specific delivery an attractive and mechanistically distinct approach. Reported strategies include IL-1Ra-releasing hydrogels, which reduced periodontal inflammation and alveolar bone absorption (and hyperglycemia) in diabetic rats [144], and other sustained-release systems designed to prolong local exposure while minimizing systemic drug levels [140,141,142,143]. Local delivery could, in principle, achieve therapeutic intratissue concentrations with a lower systemic immunosuppressive burden, and it aligns naturally with existing periodontal drug-delivery formats (gels, fibers, films). We treat local delivery as a separate translational track from systemic biologic therapy because the two differ fundamentally in pharmacokinetics, safety, regulatory path, and likely clinical indication [25,149,150,151,152].

#### 3.4.3. The Treatment Window: Timing Determines Benefit Versus Harm

An important translational lesson from the periodontal literature is that the effect of TNF-α/IL-1 blockade is time-dependent and biphasic, because these cytokines have dual roles in inflammation and in normal tissue repair. In a *Macaca mulatta* model, locally administered soluble anti-TNF-α/anti-IL-1 blockade decreased inflammatory-cell infiltration and increased apoptosis of inflammatory cells, with increased epithelial attachment and cementum formation at day 14; by day 35, however, these effects reversed, with increased inflammatory infiltration and decreased epithelial attachment and cementum formation [146]. Thus, short-term blockade facilitated periodontal wound healing, whereas prolonged blockade had adverse effects. Converging observations support this: TNF-α blockade aided early-phase wound healing after *P. gingivalis* scalp inoculation in mice (etanercept reduced fibroblast apoptosis and caspase-3 activity early [153], yet TNF-α is also required for mesenchymal stem-cell and osteoclast recruitment during fracture healing and tissue repair, so its absence impairs bone-fracture healing [154,155]. Likewise, IL-1 promotes fibroblast and keratinocyte growth, collagen synthesis, and keratinocyte chemotaxis, and facilitates healing of challenging wounds by protecting against bacterial insult [156,157]. The practical implication is that treatment timing and duration—not merely the choice of target—will determine whether cytokine blockade helps or harms the periodontium. This “treatment-window” concept deserves to be a central theme of any future translational program.

#### 3.4.4. Safety, Cost, and Patient-Selection Considerations

A balanced appraisal must weigh the risks of suppressing TNF-α or IL-1, not only the potential benefits. Systemic biologics are associated with immunosuppression and increased susceptibility to opportunistic and reactivation infections; anti-TNF-α therapy has been linked to osteonecrosis risk in the periodontally affected patients [137]; and the agents are costly, require careful patient selection and monitoring, and—crucially—lack robust randomized controlled trials conducted specifically in periodontitis. The absence of periodontitis-specific efficacy and safety trials means that current human evidence is largely indirect (from patients treated for RA or other conditions) and cannot yet establish a favorable risk–benefit balance for periodontal indications. These considerations argue for cautious, adjunctive, and ideally locally delivered use, restricted to well-characterized patient subgroups, rather than broad systemic application [158,159,160,161].

#### 3.4.5. Biomarkers for Disease Activity and Therapeutic Monitoring

TNF-α and IL-1β are not only therapeutic targets but also candidate biomarkers. Both are measurable in GCF, saliva, and serum, and their levels track with disease presence and severity [72,73,75,76]. In a precision-dentistry framework, such biomarkers could help stratify patients (identifying “high-inflammatory” phenotypes most likely to benefit from host modulation), monitor treatment response, and time the initiation or withdrawal of therapy—directly supporting the treatment-window concept above. Standardized, validated assays and prospective correlation with clinical endpoints remain needed [76,162,163,164].

#### 3.4.6. Positioning Cytokine Blockade Within the Host-Modulation Landscape

Anti-TNF-α/IL-1 strategies should be viewed alongside other host-modulation and adjunctive approaches under study, including specialized pro-resolving mediators (resolvins, lipoxins, protectins), NLRP3-inflammasome inhibitors, antioxidants and NRF2/KEAP1 pathway modulators [165,166], probiotics and oral-microbiome modulation, and regenerative strategies. Because periodontitis is multifactorial, combination approaches that pair microbial control with targeted host modulation may prove more effective than blocking any single mediator. Situating TNF-α/IL-1 blockade within this landscape guards against the oversimplified expectation that neutralizing two cytokines could control a network-driven disease on its own [24,167].

Overall, most preclinical evidence demonstrated that blocking TNF-α and IL-1β reduces inflammation and alveolar bone loss (Table 3). However, clinical evidence from periodontitis patients remains limited. The observational studies in patients receiving systemic anti-TNF-α/IL-1β therapy for inflammatory/autoimmune diseases provide indirect evidence for periodontal benefit (Table 3), but they are insufficient to establish long-term safety or efficacy when these agents are used specifically for periodontal treatment. In addition, the dual roles of TNF-α and IL-1β in inflammatory response and normal tissue repair/healing process warranted further studies on the treatment window and dosage of anti-TNF-α/IL-1β in periodontitis models.

### 3.5. Limitations of This Review

Several limitations should be acknowledged. First, much of the evidence summarized here derives from in vitro studies and animal models, whereas clinical studies of anti-TNF-α and anti-IL-1 therapy conducted specifically in periodontitis patients remain scarce; extrapolation of experimental findings to clinical practice should therefore be cautious. Second, the available studies are heterogeneous in experimental model, LPS source and species, diagnostic criteria, populations, therapeutic protocols, follow-up times, and outcomes, which limit direct comparison and generalization; the summary tables should be read with this in mind. Third, periodontitis is multifactorial: TNF-α and IL-1 are central but operate within a complex network of cytokines, chemokines, MMPs, intracellular pathways, and host–microbiome interactions, so targeting these two cytokines alone may be insufficient. Fourth, as a narrative review, this work did not apply a formal systematic- search protocol or risk-of-bias assessment, and some cited models (e.g., non-oral autoimmune models [134] differ from plaque-induced periodontitis and are included only as mechanistic or indirect context. Finally, the safety, cost, and implementation challenges of biologic therapy in dental practice are discussed here in a necessarily condensed form.

### 3.6. Future Perspectives

Future work should prioritize well-designed randomized controlled trials evaluating the efficacy and safety of anti-TNF-α and anti-IL-1 therapies as adjuncts to conventional periodontal treatment, with attention to the treatment-window concept (optimal timing, dose, and duration). Progress also depends on identifying and validating biomarkers—in GCF, saliva, and serum—that predict therapeutic response and disease progression, enabling patient stratification and biomarker-guided timing. Given the multifactorial nature of the disease, combination and multi-target strategies (cytokine networks, inflammasome activation, oxidative stress, host-immune modulation, and microbiome dysbiosis) warrant systematic exploration, as does local/controlled delivery to maximize local efficacy while minimizing systemic risk. Advances in precision dentistry—multi-omics, salivary diagnostics, and artificial-intelligence-assisted risk modeling—offer opportunities to individualize therapy. Finally, longitudinal studies integrating clinical, molecular, microbiological, and immunological data, together with evaluation of long- term safety and cost-effectiveness relative to conventional and emerging host-modulation strategies, will be essential to translate immunomodulatory therapy into routine care.

## 4. Conclusions

This review highlights how LPS-initiated signaling promotes TNF-α and IL-1 production and how these cytokines amplify RANKL-driven osteoclastogenesis to couple inflammation with alveolar bone loss in periodontitis. Several points frame the translational outlook. First, periodontitis is a multifactorial, dysbiosis-driven disease arising from the interplay of the subgingival biofilm, host immune response, and genetic and environmental factors; LPS is one important stimulus among many, and TNF-α and IL-1 act within a broader inflammatory network. Second, preclinical evidence consistently shows that TNF-α and IL-1 blockades reduce periodontal inflammation and bone loss, but the dual roles of these cytokines in inflammation and repair make treatment timing decisive—short-term blockade can aid healing while prolonged blockade can impair it. Third, cytokine blockade is best positioned as an adjunct to, not a replacement for, mechanical biofilm control, and given systemic-safety concerns, local/targeted delivery is a particularly promising route. Fourth, most current evidence is preclinical or indirect, and the number of high-quality clinical studies in periodontitis patients is still limited; conclusions should be calibrated accordingly. The principal open questions for translation are therefore the optimal delivery route, treatment timing and window, the role of concurrent microbial control, the safety of cytokine suppression, and biomarker-guided patient selection—resolving these is a prerequisite for meaningful clinical trials of anti-TNF-α/IL-1 therapy in periodontitis.

## Figures and Tables

**Table 1:** Cytokine changes and subsequent effects after in-vivo LPS exposure.

Model	LPS Source	LPS Exposure Regimen	Cytokine Changes	Phenotypic Changes	References
Rat	*P. g*-LPS	Periodontal pocket injection	↑ TNF-α, IL-1β, IL-6	PD and ABL	[57]
Rat	*E. coli*-LPS	Palatal tissues injection	↑ NF-κB and RankL	PD and ABL	[58]
Rat	*E. coli*-LPS	Gingival sulcus topical application	↑ TNF-α	↑ OCs	[64]
Rat	Not specified	Intraperitoneal injection with ligature placement	↑ TNF-α, IL-6, NLRP3, cleaved-caspase-1, and RANKL	PD and ABL	[59]

*P. g*-LPS: *P. gingivalis*-LPS; PD: periodontitis; ABL: alveolar bone loss; OCs: osteoclasts; ↑: increase.

**Table 2:** Cytokine changes and subsequent effects after in-vitro LPS exposure.

Cell Type	LPS Source	Cytokine Changes	Phenotypic Changes	References
MBMMs	Not specified	N/A	↑ TRAP-positive OCs	[65]
MBSCL and MBMCs	*E. coli*-LPS	↑ TNF-α, IL-1β, IL-6, and sRANKL	↑ TRAP-positive OCs	[64]
MBMM	*P. g*-LPS	↑ TNF-α, IL-6, iNOS, and COX2	↑ OC formation and bone resportion	[66]
MMCL	*P. g*-LPS	↑ TNF-α, IL-1β, IL-6, and MMP9	N/A	[67]
MMCL	Not specified	↑ TNF-α, iNOS, IL-6, NLRP3, GSDMD, cleaved-IL-1β, and cleaved-caspase-1	M1 Mφ polarization	[60]
MBMMs	*E. coli*-LPS	↑ TNF-α, IL-1β, IL-6	↑ TRAP-positive OCs	[68]
MMCL	*E. coli*-LPS	↑ TNF-α, IL-1β, IL-6	M1 Mφ polarization	[69]
HMCL	*P. g*-LPS	↑ TNF-α, IL-1β, IL-6	M1 Mφ polarization	[61]
hPDLSCs	*P. g*-LPS	↑ TNF-α, IL-1β, IL-6, COX2, and iNOS	↓ Osteogenesis	[62]
hPDLCs	Not specified	↑ TNF-α, IL-6, iNOS, COX2, and ROS	↓ ALP-positive cells	[63]
MDCs and T cells	Not specified	↑ TNF-α, IL-1β	↑ Inflammatory immune response	[70]

MBMMs: mouse bone marrow macrophages; OCs: osteoclasts; MBSCL: mouse bone-marrow stromal cell line; MBMC: mouse bone marrow cells; MMCL: mouse macrophage cell line; Mφ: Macrophage; HMCL: human macrophage cell line; hPDLSCs: human periodontal ligament stem cells; hPDLCs: human periodontal ligament cells; MDCs: mouse dendritic cells; ↑: increase; ↓: decrease.

**Table 3:** The effects of anti-TNF-α and anti-IL-1β intervention on periodontal tissues.

Subject	Periodontitis Model	Therapeutic Reagents	Therapeutic Effect	References
Mφs	*A. ctinomycetemcomitans* LPS administration	N/A	IL-1Ra KO: ↑ IL-1α, IL-1β, TNF-α, IL-6 ↑ RANK, OC formation, ↑ Cox2, PGE2, Ep4	[139]
hPLFs & pBMCs	N/A	Anakinra	↓ OC formation ↓ IL-1β induced leukocyte clusters ↓ RANKL/OPG No effect on osteogenesis	[138]
C57/BL6J mice	Ligature-induced periodontitis and Imiquimod (IMQ)-induced psoriasis	Periodontal therapy + Adalimumab	↓ Serum IL-6 and IL-17A ↓ CEJ–ABC ↓ alveolar bone loss	[144]
*FcγRIIb^−/−^* mice	Mandibular bone loss present	Etanercept	↓ Mandibular bone loss ↑ Osx, Alp, and Ocn	[134]
Wistar rats	Ligature placement	Infliximab	↓ Granulocyte blood counts ↓ Gingival IL-1b, TNF-α and MPO levels ↑ Periodontal histopathological scores ↑ Collagen network	[133]
STZ-induced diabetic rats	Ligature placement	IL-1Ra-releasing Hydrogel	↓ Periodontal inflammation ↓ Alveolar bone absorption ↓ Hyperglycemia	[145]
Nonhuman primate	Ligature + Open flap periodontal surgery	sIL-1RI + sTNFR:Fc	Day 14: ↓ Inflammatory cells ↓ OC formation ↑ Epithelial attachment ↑ Cementum formation Day 35: The above effects were all reversed	[146]
RA patients	Existing periodontitis	Infliximab	↓ Attachment loss ↑ Gingivitis which was not associated with more periodontal disease	[135]
RA patients	N/A	Infliximab	↓ Bleeding on probing ↓ Probing depth ↓ Clinical attachment loss ↓ TNF-α levels in the GCF	[147]
RA patients	N/A	Adalimumab	↓ Gingival index ↓ Bleeding on probing ↓ Probing depth ↓ Serum C-reactive protein ↓ Serum IL-6 ↓ Serum TNF-α	[148]
RA patients	Existing periodontitis	Anti-TNF-α	↑ the effectiveness of non-surgical periodontal therapy	[136]

Mφs: macrophages; IL-1Ra: IL-1 receptor antagonist; OC: osteoclast; hPLFs: human periodontal ligament fibroblasts; pBMCs: peripheral blood mononuclear cells; RA: rheumatoid arthritis; *FcγRIIb^−/−^* mice: a mouse model for systemic lupus erythematosus; CEJ–ABC: cementoenamel junction–alveolar bone crest distance; STZ: Streptozotocin; anti-TNF-α agents: Etanercept, Infliximab & Adalimumab; anti-IL-1 agents: Anakinra; ↑: increase; ↓: decrease.

## Data Availability

No new data were created or analyzed in this study. Data sharing is not applicable to this article.
